# Targeting the pattern‐triggered immunity pathway to enhance resistance to *Fusarium graminearum*

**DOI:** 10.1111/mpp.12781

**Published:** 2019-02-06

**Authors:** Sujon Sarowar, Syeda T. Alam, Ragiba Makandar, Hyeonju Lee, Harold N. Trick, Yanhong Dong, Jyoti Shah

**Affiliations:** ^1^ Department of Biological Sciences University of North Texas Denton TX 76201 USA; ^2^ BioDiscovery Institute University of North Texas Denton TX 76201 USA; ^3^ Department of Plant Sciences University of Hyderabad Gachibowli Hyderabad 500046 India; ^4^ Department of Plant Pathology Kansas State University Manhattan KS 66506 USA; ^5^ Department of Plant Pathology University of Minnesota St. Paul MN 55108 USA; ^6^ Present address: Botanical Genetics Buffalo NY USA

**Keywords:** *Arabidopsis thaliana*, flg22 peptide, Fusarium head blight, microbe‐associated molecular pattern, PTI, wheat, *WRKY29*

## Abstract

Fusarium head blight (FHB) is a disease of the floral tissues of wheat and barley for which highly resistant varieties are not available. Thus, there is a need to identify genes/mechanisms that can be targeted for the control of this devastating disease. *Fusarium graminearum* is the primary causal agent of FHB in North America. In addition, it also causes Fusarium seedling blight. *Fusarium graminearum* can also cause disease in the model plant *Arabidopsis thaliana*. The Arabidopsis–*F. graminearum* pathosystem has facilitated the identification of targets for the control of disease caused by this fungus. Here, we show that resistance against *F. graminearum* can be enhanced by flg22, a bacterial microbe‐associated molecular pattern (MAMP). flg22‐induced resistance in Arabidopsis requires its cognate pattern recognition receptor (PRR) FLS2, and is accompanied by the up‐regulation of *WRKY29*. The expression of *WRKY29*, which is associated with pattern‐triggered immunity (PTI), is also induced in response to *F. graminearum* infection. Furthermore, *WRKY29* is required for basal resistance as well as flg22‐induced resistance to *F. graminearum*. Moreover, constitutive expression of *WRKY29* in Arabidopsis enhances disease resistance. The PTI pathway is also activated in response to *F. graminearum* infection of wheat. Furthermore, flg22 application and ectopic expression of *WRKY29* enhance FHB resistance in wheat. Thus, we conclude that the PTI pathway provides a target for the control of FHB in wheat. We further show that the ectopic expression of *WRKY29* in wheat results in shorter stature and early heading time, traits that are important to wheat breeding.

## Introduction

The ascomycetous fungus *Fusarium graminearum* (hereafter referred to as *Fg*) is an important phytopathogen. In wheat (*Triticum aestivum*) and barley (*Hordeum vulgare*), *Fg* is the primary causal agent of Fusarium head blight (FHB) disease which affects floral tissues (Bai and Shaner, 2004; McMullen *et al*., 1997a; Xu and Nicholson, 2009). In addition, it also causes Fusarium seedling blight. FHB epidemics in the past have resulted in $0.3–3 billion in losses (Bai and Shaner, 2004; Johnson *et al*., 2003; Wilson *et al*., 2017). FHB adversely impacts grain yield and quality. Mycotoxins, for example deoxynivalenol (DON), which accumulate in infected grains, further limit grain acceptability for human and animal consumption (Bai and Shaner, 2004; McMullen *et al*., 1997b; Wilson *et al*., 2017). Monogenic gene‐for‐gene‐type resistance is not available for FHB. In many cultivated wheat varieties, resistance to FHB is derived from the cultivar Sumai 3 and its derivatives (Bai and Shaner, 2004). Sumai 3‐derived resistance is a quantitative trait that limits fungal spread from the infection site. The non‐availability of highly resistant wheat and barley cultivars, the practical difficulties with the timing of fungicide application during anthesis and the high humidity conditions when disease threat is the highest further constrain efforts to control FHB (McMullen *et al*., 1997b; Pirgozliev *et al*., 2003).

The genes and mechanisms that contribute to the basal resistance to *Fg* offer targets for molecular breeding and genetic engineering of FHB resistance. For example, salicylic acid (SA) signalling, which contributes to basal resistance to FHB in wheat and barley (Diethelm *et al*., 2014; Hao *et al*., 2018; Makandar *et al*., 2006, 2012, 2015), is a target for enhancing FHB resistance. FHB resistance in wheat was enhanced by the constitutive expression of *NPR1* (*NON‐EXPRESSOR OF PR GENES 1*), which is a key regulator of SA signalling, and *NPR1*‐like genes in wheat (Gao CS *et al*., 2013; Makandar *et al*., 2006; Yu *et al*., 2017). Furthermore, natural variations at two homeologous *NPR1*‐like genes located on the long arm of chromosomes 2A and 2D were associated with resistance to FHB in winter wheat (Diethelm *et al*., 2014). FHB resistance was also enhanced in transgenic wheat that accumulated higher levels of SA as a result of the constitutive expression of *PAD4*, a positive modulator of SA accumulation (Makandar *et al*., 2015). FHB resistance was also enhanced in barley plants that overexpressed *ICS*, a gene that encodes an isochorismate synthase, which synthesizes SA (Hao *et al*., 2018). In contrast, RNA interference (RNAi)‐mediated repression of *ICS* in barley compromised FHB resistance (Hao *et al*., 2018).

Pattern‐triggered immunity (PTI) is another process that can be targeted to promote disease resistance. PTI, which involves a complex set of physiological and molecular responses in the plant, including reactive oxygen species (ROS) accumulation and callose deposition, is induced in response to the recognition of conserved microbe‐associated molecular patterns (MAMPs) by pattern recognition receptors (PRRs) located on the plant cell surface (Bigeard *et al*., 2015; Li *et al*., 2016). PTI is an important contributor to non‐host resistance in plants (Bigeard *et al*., 2015). Some well‐studied MAMPs include the bacterial flagellar protein flagellin and elongation factor EF‐Tu, which are perceived by the cognate PRRs FLS2 (FLAGELLIN‐SENSITIVE 2) and EFR (EF‐Tu RECEPTOR), respectively (Bigeard *et al*., 2015). A 22‐amino‐acid long region of flagellin, epitomized by flg22 from *Pseudomonas aeruginosa*, is sufficient for the activation of PTI via *FLS2* (Gomez‐Gomez and Boller, 2000), whereas an 18‐amino‐acid long epitope of EF‐Tu, represented by elf‐18 from *Escherichia coli*, is sufficient for PTI activation through *EFR* (Zipfel *et al*., 2006). The polysaccharide chitin, which is a major component of fungal cell walls, is another MAMP (Sánchez‐Vallet *et al*., 2015). In *Arabidopsis thaliana*, LysM (extracellular lysin motifs)‐containing receptor‐like kinases have been implicated in chitin signalling and resistance against fungal pathogens (Sánchez‐Vallet *et al*., 2015; Wan *et al*., 2008). Similarly, in rice (*Oryza sativa*, Os), chitin fragments are perceived by the LysM domain‐containing OsCEBiP (chitin elicitor binding protein) and OsCERK1 (Kaku *et al*., 2006; Shimizu *et al*., 2010). In barley, the LysM domain‐containing HvCERK1 (Chitin Elicitor Receptor Kinase 1) is required for plant response to chitin (Karre *et al*., 2017). Wheat leaves are also responsive to chitin and flg22, both of which induce the expression of wheat homologues of chitin‐ and flg22‐responsive Arabidopsis genes, including *TaPUB23*‐like and *TaWRKY23*‐like (Schoonbeek *et al*., 2015). In addition, the expression of Arabidopsis *EFR* is sufficient to confer elf‐18 recognition and to enhance resistance in wheat against the bacterial pathogen *Pseudomonas syringae* pv. *oryzae* (Schoonbeek *et al*., 2015), therefore suggesting the conservation of PTI signalling mechanisms between Arabidopsis and wheat.

There is significant overlap in the genes that are up‐regulated by different MAMPs (Gust *et al*., 2007; Wan *et al*., 2008; Zipfel *et al*., 2006), thus signifying the activation of convergent signalling pathways by these discrete MAMPs, which control the expression of a common set of PTI‐associated genes, although with different dynamics and amplitudes (Li *et al*., 2016). In Arabidopsis, *WRKY29*, which encodes a WRKY family transcription factor, is one such gene that is up‐regulated by both flg22 and chitooligosaccharide (Asai *et al*., 2002; Wan *et al*., 2008). This convergence of signalling associated with different MAMPs has led to the suggestion that MAMPs are perceived as general danger signals and that plants do not distinguish between different microbes via the defence signalling induced by different MAMPs (Zipfel *et al*., 2006). Therefore, it is expected that PTI activation should confer cross‐protection against pathogens in different kingdoms. Indeed, ectopic application of the bacterial MAMP, flg22, enhances resistance in Arabidopsis to the fungal pathogen *Botrytis cinerea* (Ferrari *et al*., 2007; Galletti *et al*., 2011). Moreover, the application of chitooligosaccharide promotes resistance in Arabidopsis to the bacterial pathogen *Pseudomonas syringae* pv. *tomato* DC3000 (Wan *et al*., 2008). Cross‐protection also extends to *Fg*. Chaturvedi *et al*. (2012) showed that prior treatment with a bacterial pathogen promotes resistance against *Fg* in Arabidopsis, which has been utilized in several studies as a model plant to characterize the physiological and molecular aspects of plant defence against *Fg* (Chen *et al*., 2006, 2009; Cuzick *et al*., 2008; Makandar *et al*., 2006, 2010, 2015; Nalam *et al*., 2015; Savitch *et al*., 2007; Skadsen and Hohn, 2004; Urban *et al*., 2002; Van Hemelrijck *et al*., 2006). *Fg* can infect leaves and inflorescences of Arabidopsis.

The PTI pathway has been implicated as a major player in the resistance to *Fusarium* ear rot in the maize inbred line BT‐1 (Wang *et al*., 2016). Similarly, basal resistance to FHB in barley requires HvCERK1 (Karre *et al*., 2017), thus suggesting that the PTI pathway is engaged during *Fg* infection. The aim of this study was to determine whether PTI can be targeted to enhance resistance against *Fg. *We show that Arabidopsis can be protected against *Fg* infection by flg22‐mediated induction of PTI via *FLS2*. This resistance to *Fg* infection conferred by flg22 requires *WRKY29* which, when constitutively expressed in Arabidopsis, confers a high level of resistance to *Fg*. We further demonstrate that flg22 application and constitutive expression of Arabidopsis *WRKY29* confer enhanced resistance to FHB in wheat, which is accompanied by stronger expression of PTI‐associated genes, thus supporting our suggestion that the PTI pathway is a target for enhancing resistance to FHB.

## Results

### *Fg* infection induces *WRKY29 *expression in *A. thaliana*

**Figure 1:**
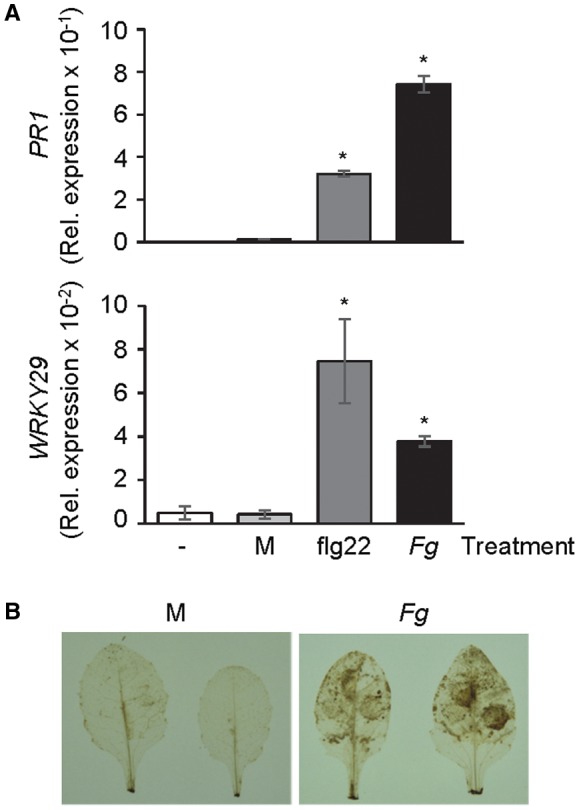
Induction of pattern‐triggered immunity (PTI) markers in flg22‐treated and *Fusarium graminearum *(*Fg*)‐inoculated Arabidopsis leaves. (A) *PR1 *and *WRKY29 *expression, relative to At1g07940, in flg22‐treated and *Fg*‐inoculated plants at 24 h post‐treatment. Top: real‐time reverse transcription‐polymerase chain reaction (RT‐PCR) analysis of *PR1* expression in leaves of wild‐type Arabidopsis accession Columbia plants infiltrated with 50 ng flg22 peptide and in plants inoculated with *Fg*. Untreated and mock (M)‐inoculated plants provided the controls. Bottom: real‐time RT‐PCR analysis of *WRKY29* expression in the above samples. Error bars represent the standard error (SE) (*n* = 5). Asterisks above the bars indicate values that are significantly different (*P* < 0.05; *t*‐test) from the mock‐inoculated plants. (B) 3,3′‐Diaminobenzidine (DAB) staining to monitor H_2_O_2_ accumulation in mock (M)‐ and *Fg*‐inoculated Arabidopsis leaves. Leaves were stained with DAB at 18 h post‐inoculation. Brown deposits indicate H_2_O_2_ accumulation.

The expression of *WRKY29* was used as a molecular marker of PTI to test whether *Fg* infection induces a PTI‐like mechanism in Arabidopsis. *Fg* was infiltrated into Arabidopsis leaves and *WRKY29* expression was monitored by real‐time reverse transcription‐polymerase chain reaction (RT‐PCR). flg22 peptide‐treated leaves provided the positive control for *WRKY29 *expression. In addition, expression of the *Fg‐* and flg22‐responsive *PATHOGENESIS‐RELATED 1 *(*PR1*) (Asai *et al*., 2002; Makandar *et al*., 2006; Yi *et al*., 2014) was monitored as a positive control for the two treatments. Expression of *PR1*, which encodes a cysteine‐rich secretory protein, has been used as an excellent molecular marker for the activation of SA signalling in plants. As shown in Fig. 1A, *PR1 *and *WRKY29 *expression were up‐regulated in *Fg‐* and flg22‐treated leaves compared with the untreated and mock‐inoculated controls, thus confirming the activation of downstream signalling by these treatments. *Fg* infection also resulted in the accumulation of hydrogen peroxide (H_2_O_2_), another hallmark of PTI (Fig. 1B). Taken together, these results suggest that Arabidopsis responds to *Fg* infection by stimulating a PTI‐like response.

### The flg22 peptide induces resistance against *Fg* infection in *A. thaliana* and wheat

**Figure 2:**
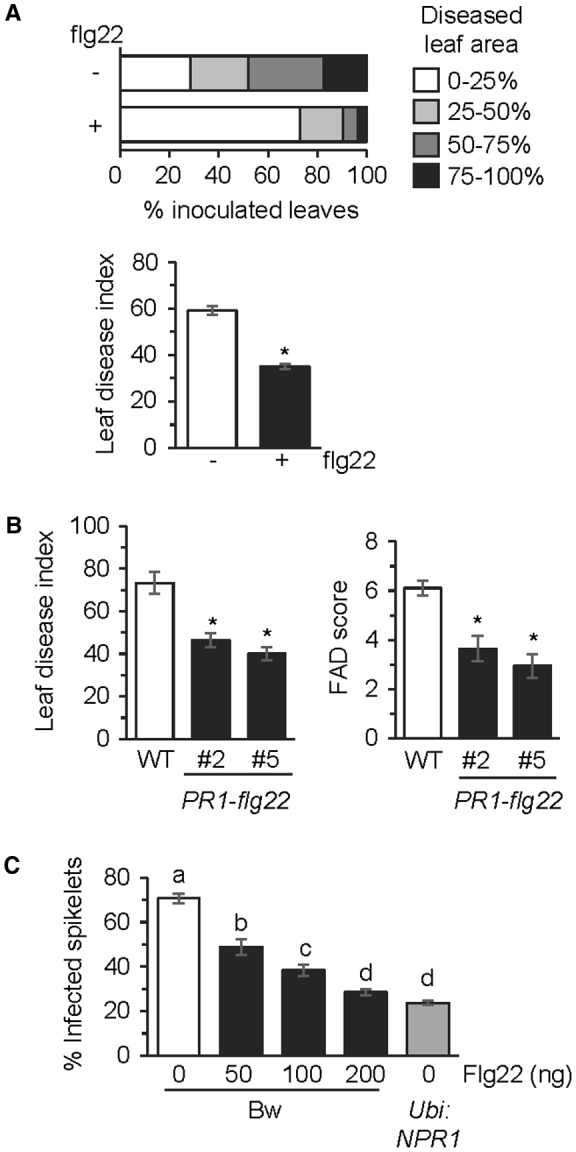
flg22 application enhances resistance to *Fusarium graminearum *in Arabidopsis and wheat. (A) Top: *F. graminearum *disease severity in wild‐type (WT) Arabidopsis accession Columbia leaves treated with 50 ng of flg22 (+) and as control with water (–). Fungal inoculation was conducted 24 h after flg22 treatment and disease severity was monitored at 5 days post‐inoculation (dpi) with the fungus (*n* = 50). Bottom: leaf disease index in the above experiment. All values are means ± standard error (SE) (*n* = 50). Asterisks above the bars indicate values that are significantly different from the water‐treated control plants (*P* < 0.05; *χ* ^2^ test). (B) Left: leaf disease index in *F. graminearum*‐inoculated WT accession Columbia plant and two independent *PR1‐flg22* transgenic lines that are in the *FLS2* background. All values are the means ± SE (*n* = 50). Asterisks above the bars indicate values that are significantly different from WT (*P* < 0.05; *χ* ^2^ test). Right: Fusarium Arabidopsis Disease (FAD) score in inflorescences of the WT and *PR1‐flg22* lines in the *FLS2* background. All values are the means ± SE (*n* = 30). Asterisks above the bars indicate values that are significantly different from WT (*P* < 0.05; *t*‐test). (C) Fusarium head blight severity in flg22‐treated wheat cv. Bobwhite (Bw). The spikelets were treated with the indicated amounts of flg22 peptide at 24 h prior to fungal inoculation. The *Ubi:NPR1* transgenic plant, which is in the cultivar Bw background, provided the FHB‐resistant control. Disease severity was monitored at 21 dpi. All values are the means ± SE (*n* = 10). Different letters above the bars indicate values that are significantly different from each other (*P* < 0.05; Tukey’s test).

To determine whether the PTI pathway can be targeted to enhance resistance against *Fg*, we tested whether pretreatment of Arabidopsis leaves with the flg22 peptide is capable of augmenting resistance to *Fg*. Leaves of wild‐type (WT) Arabidopsis accession Columbia plants were infiltrated with flg22 peptide to activate PTI; 24 h later, the same leaves were inoculated with *Fg* and disease severity was scored at 5 days post‐inoculation (dpi). As shown in Fig. 2A, *Fg* disease severity was significantly lower in flg22‐treated leaves than in mock‐treated leaves, thus suggesting that an flg22‐activated mechanism can enhance resistance against *Fg*. We further tested basal resistance to *Fg* in transgenic Arabidopsis engineered to express a chimeric *PR1‐flg22* construct that expresses flg22 fused to the C‐terminus of PR1. As mentioned above, PR1 is a secretory protein that accumulates in the apoplast (Gu and Innes, 2012; Pečenková *et al*., 2017; Watanabe *et al*., 2013). Furthermore, the activation of SA signalling promotes the export of PR1 into the apoplast (Wang *et al*., 2005). Thus, the PR1‐flg22 fusion is expected to deliver flg22 into the apoplast, where it should be perceived by FLS2 to activate PTI. As shown in Fig. 2B, *Fg* disease severity was lower in leaves of two independently derived *PR1‐flg22*‐expressing transgenic lines compared with the WT control (Figs 2B and S1, see Supporting Information). Disease severity was also lower in the inflorescence of *PR1‐flg22* lines compared with the WT control (Fig. 2B). These results confirm that an flg22‐activated mechanism can confer resistance to *Fg* infection in Arabidopsis.

We further tested whether flg22 application was capable of promoting FHB resistance in wheat. Varying amounts of the flg22 peptide dissolved in 10 µL of water were applied with a syringe to two central spikelets of each spike of the spring wheat cultivar Bobwhite; 24 h later, these spikelets were inoculated with *Fg *and FHB disease severity was monitored 21 days later. A *Ubi*:*NPR1* wheat line, which is in the Bobwhite background and constitutively expresses the Arabidopsis *NPR1* gene from the maize *Ubiquitin* promoter to increase FHB resistance (Makandar *et al*., 2006, 2012), provided the disease‐resistant control for this experiment. As shown in Fig. 2C, pretreatment with flg22 peptide enhanced FHB resistance in the wheat cultivar Bobwhite. The resistance‐promoting effect of flg22 exhibited a dose‐dependent response. At the highest level of 200 ng, the FHB resistance‐promoting effect of flg22 was comparable with that observed in the *Ubi*:*NPR1* line. Taken together, these experiments with Arabidopsis and wheat signify the potential for targeting PTI to enhance resistance against *Fg*.

### *FLS2* is required for flg22‐induced resistance to *Fg* infection in *A. thaliana*

**Figure 3:**
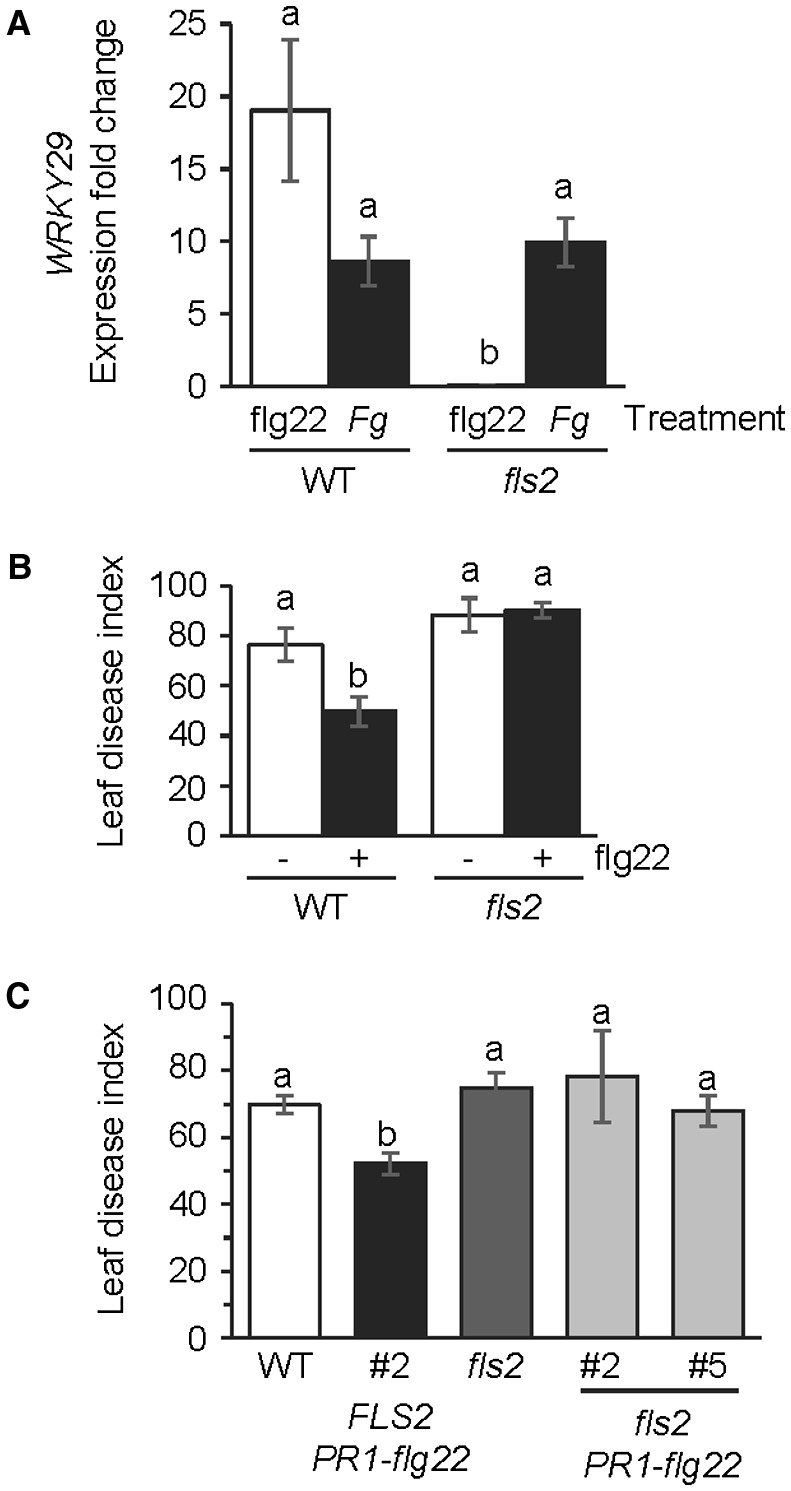
*FLS2* function is essential for the flg22‐induced resistance to *Fusarium graminearum *(*Fg*) in Arabidopsis. (A) Real‐time reverse transcription‐polymerase chain reaction (RT‐PCR) evaluation of fold induction of *WRKY29* expression in flg22‐treated and *Fg*‐inoculated leaves of wild‐type (WT) and *fls2* mutant plants, relative to expression in the corresponding mock‐inoculated leaves. Gene expression was monitored 24 h post‐treatment, with the expression of At1g07940 providing the control. All values are the means ± standard error (SE) (*n* = 5). Different letters above the bars indicate values that are significantly different from each other (*P *< 0.05; Tukey’s test). (B) Leaf disease index in WT accession Columbia and *fls2* mutant leaves treated with 50 ng of flg22 (+) or as control with water (–). Fungal inoculation was conducted 24 h after flg22 treatment and disease severity was monitored at 5 days post‐inoculation (dpi) with the fungus. All values are the means ± SE (*n* = 50). Different letters above the bars indicate values that are significantly different from each other (*P* < 0.05; Tukey’s test). (C) Leaf disease index in *Fg*‐inoculated WT accession Columbia plant, *PR1‐flg22* transgenic line #2 in the *FLS2* background, the *fls2* mutant and two independent *PR1‐flg22* transgenic lines in the *fls2 *mutant background. Disease severity was monitored at 5 dpi. All values are the means ± SE (*n* = 50). Different letters above the bars indicate values that are significantly different from each other (*P* < 0.05; Tukey’s test).

To confirm that the flg22‐induced resistance to *Fg* was indeed a result of the activation of PTI, the ability of flg22 to enhance resistance to *Fg* in the *fls2* mutant was studied. *WRKY29* expression was monitored as a molecular marker for the activation of PTI. As shown in Fig. 3A, although flg22 treatment, compared with mock treatment, was effective in inducing *WRKY29 *expression in the WT plant, flg22 was unable to induce *WRKY29* expression in the *fls2* mutant, thus confirming the requirement of *FLS2* for the flg22‐induced expression of *WRKY29*. Compared with the WT, the *Fg *resistance‐promoting effect of flg22 was not observed in the *fls2* mutant (Fig. 3B). The *Fg *disease severity in leaves of the flg22‐treated *fls2* mutant was comparable with that in the mock‐treated *fls2* mutant and significantly higher than that in flg22‐treated WT plants. Experiments with the *PR1‐flg22* chimera also confirmed the importance of *FLS2* to flg22‐induced resistance to *Fg*, which was lacking in the *fls2 *mutant background compared with the *FLS2* background (Fig. 3C).

Although the leaf disease index, which reflects the average disease severity across the different disease categories (see Experimental procedures), was not significantly different between the WT and *fls2* plants that were not treated with flg22 (Fig. 3B,C), we repeatedly observed significant differences (*P* < 0.05; *χ* ^2^ test) in the distribution of the four disease categories in the *fls2* mutant compared with the WT. We therefore suggest that there is a subtle influence of the *FLS2* allele on the basal resistance to *Fg* in Arabidopsis.

### flg22‐induced resistance to *Fg* in *A. thaliana* requires *NPR1* and *WRKY29*

**Figure 4:**
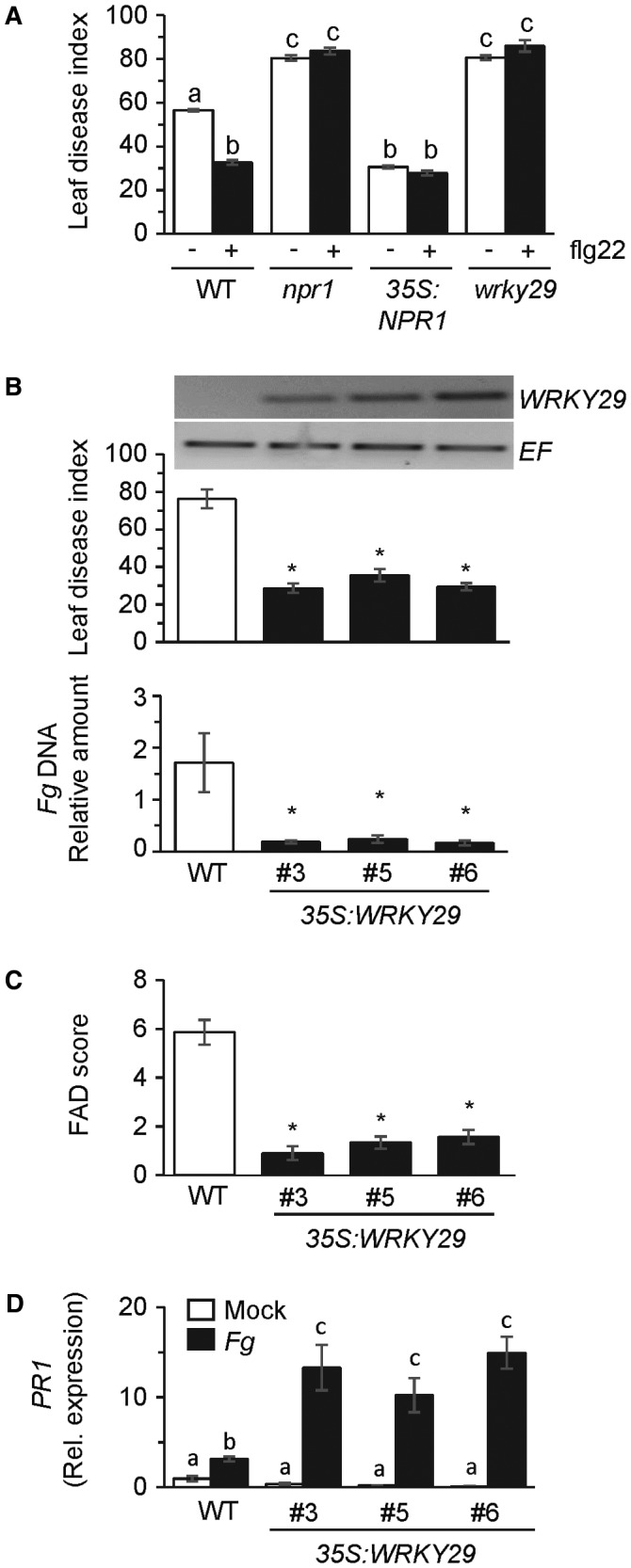
Constitutive expression of *WRKY29 *in Arabidopsis promotes resistance to *Fusarium graminearum* (*Fg*). (A) Leaf disease index in *Fg*‐inoculated wild‐type (WT) accession Columbia, the *npr1* mutant, a *35S:NPR1* transgenic line in which the *NPR1* coding sequence is expressed from the *35S* promoter, and a *wrky29* mutant. Leaves were treated with 50 ng of flg22 peptide, or mock treated, 24 h prior to fungal inoculation. Disease was monitored at 5 days post‐inoculation (dpi). All values are the means ± standard error (SE) (*n* = 50). Different letters above the bars indicate values that are significantly different from each other (*P* < 0.05; Tukey’s test). (B) Top: reverse transcription‐polymerase chain reaction (RT‐PCR) demonstration of *WRKY29* expression and, as control, At1g07940 (EF) expression in leaves of WT accession Columbia and three independent *35S:WRKY29* lines in which the *WRKY29* coding sequence is expressed from the *35S* promoter. Middle: leaf disease index in WT accession Columbia and *35S:WRKY29* lines inoculated with *Fg. *Disease was monitored at 5 dpi. All values are the means ± SE (*n* = 50). Asterisks above the bars indicate values that are significantly different from the WT (*P* < 0.05; *χ* ^2^ test). Bottom: real‐time PCR analysis (×10^−2^) of DNA content of *Fg nahG *gene relative to the *Arabidopsis ACT8* gene. All values are means ± SE (*n = *4) in leaves of WT Col‐0 and the *35S:WRKY29 *plants at 4 dpi with *Fg*. Asterisks above the bars indicate values that are significantly different from the WT (*P* < 0.05; *t*‐test). (C) Fusarium Arabidopsis Disease (FAD) score for the WT accession Columbia and *35S:WRKY29* transgenic lines. All values are the means ± SE (*n* = 30). Asterisks above the bars indicate values that are significantly different from the WT (*P* < 0.05; *t*‐test). (D) Real‐time RT‐PCR evaluation of *PR1* expression in mock‐ and *Fg*‐inoculated leaves of WT accession Columbia and *35S:WRKY29* transgenic lines. Gene expression relative to expression of the control gene At1g07940 was monitored at 24 h post‐treatment. All values are the means ± SE (*n* = 4). Different letters above the bars indicate values that are significantly different from each other (*P* < 0.05; Tukey’s test).

SA signalling has an important function in Arabidopsis and wheat defence against *Fg* (Diethelm *et al*., 2014; Gao CS *et al*., 2013; Makandar *et al*., 2006, 2010, 2012, 2015; Yu *et al*., 2017). In Arabidopsis, SA signalling is also induced in response to flg22 (Tsuda *et al*., 2008; Yi *et al*., 2014). Furthermore, SA stimulates *FLS2* expression and SA analogues prime the induction of flg22‐triggered responses, including flg22‐triggered up‐regulation of *WRKY29* expression (Pick *et al*., 2012; Yi *et al*., 2014). In contrast, flg22‐triggered responses, including *WRKY29 *expression, are attenuated in the SA biosynthesis *sid2* mutant (Yi *et al*., 2014). To determine whether SA signalling is critical for the flg22‐conferred resistance to *Fg*, we tested the ability of flg22 to promote resistance to *Fg* in the SA‐insensitive *npr1* mutant. 35S:*NPR1* plants, which constitutively express *NPR1* from the *Cauliflower mosaic virus* *35S* promoter (Cao *et al*., 1998; Makandar *et al*., 2006), provided the *Fg*‐resistant control for this experiment. As shown in Fig. 4A and reported previously (Makandar *et al*., 2010), *Fg* disease severity was higher in leaves of the *npr1* mutant than in the WT plant. Furthermore, flg22 was unable to enhance resistance to *Fg* in the *npr1* mutant compared with the WT, thus confirming that flg22‐induced PTI cannot bypass the need for SA signalling in defence against *Fg*. Similarly, *WRKY29* function was required for defence against *Fg* (Fig. 4A). Compared with the WT, *Fg* disease severity was higher in the *wrky29* mutant and comparable with that in the *npr1* mutant. Furthermore, unlike in the WT, flg22 was unable to promote resistance in the *wrky29* mutant. These results provide further confirmation that the flg22‐conferred resistance to *Fg* is mediated through genetic components that function downstream of the *FLS2*/flg22 receptor/ligand pair. The higher level of disease in the *wrky29* mutant compared with the WT further indicates that a *WRKY29*‐dependent defence mechanism(s) is critical for basal resistance to *Fg*.

### Constitutive expression of *WRKY29* enhances resistance to *Fg* in *A. thaliana*

We further tested the feasibility of engineering the PTI pathway to enhance resistance against *Fg* by developing plants that constitutively express *WRKY29*, which encodes a transcription factor that is common to PTI induced by flg22 and chitin (Asai *et al*., 2002; Wan *et al*., 2008). The *Cauliflower mosaic virus 35S* promoter was used to constitutively express *WRKY29* in Arabidopsis (Fig. 4B). Compared with the WT plant, *Fg* disease severity and fungal accumulation, which was monitored by comparing the accumulation of *Fg NahG* gene DNA, were significantly lower in leaves of all three independently derived *35S*:*WRKY29* plants (Fig. 4B). Similarly, *Fg* disease severity was also lower in the inflorescence tissues of *35S*:*WRKY29* plants compared with the WT (Fig. 4C). These results provide further proof‐of‐concept that the PTI pathway is amenable for engineering resistance to *Fg*. Compared with the WT, basal expression of the SA‐ and flg22‐responsive *PR1* gene was not altered in *35S*:*WRKY29* plants (Fig. 4D). However, fungal infection resulted in significantly stronger induction of *PR1 *in *35S*:*WRKY29* plants than in the WT (Fig. 4D), therefore indicating that constitutive expression of *WRKY29* promotes robust activation of defence responses. In contrast, constitutive expression of *WRKY29* did not result in stronger accumulation of H_2_O_2_ in response to fungal infection (Fig. S2A, see Supporting Information).

### Wheat engineered to express *WRKY29* exhibits enhanced resistance to FHB and seedling blight

**Figure 5:**
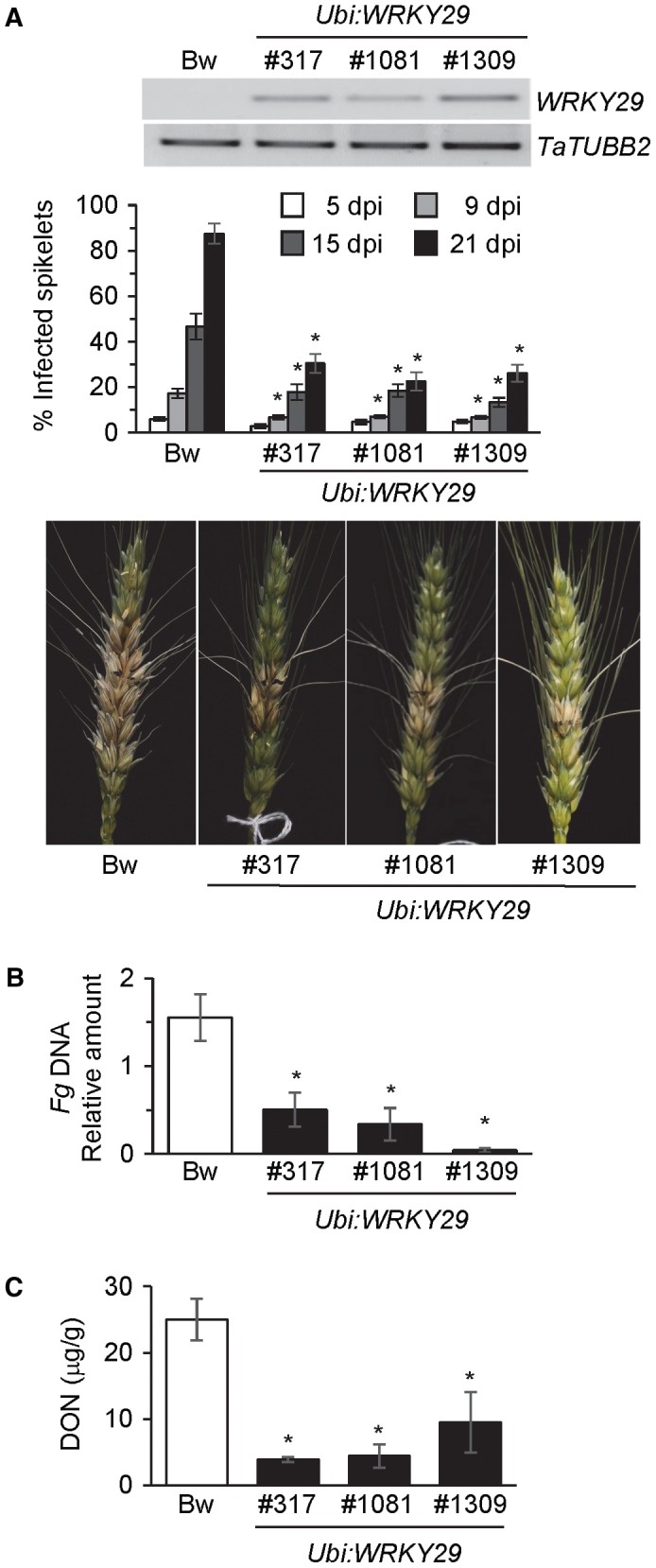
Ectopic expression of *WRKY29 *in wheat promotes resistance to Fusarium head blight. (A) Top: reverse transcription‐polymerase chain reaction (RT‐PCR) demonstration of *WRKY29* expression in leaves of wheat cv. Bobwhite (Bw) and three independent *Ubi:WRKY29* transgenic wheat lines in which the Arabidopsis *WRKY29* coding sequence is expressed from the maize *Ubi* promoter. Middle: FHB disease severity in wheat cv. Bw and *Ubi:WRKY29* lines in the Bw background. Disease progression was monitored at 5, 9, 15 and 21 days post‐inoculation (dpi) of spikes. All values are the means ± standard error (SE) (*n* = 12). Asterisks above the bars indicate values that are significantly different from the WT for that particular time point (*P* < 0.05; *t*‐test). Bottom: photograph showing disease spread in a representative spike from wheat cv. Bw and the *Ubi:WRKY29* lines. Photographs were taken at 21 dpi. (B) Real‐time PCR analysis of the DNA content of the *Fusarium graminearum* (*Fg*) *NahG *gene relative to the wheat *TUB2* gene in wheat spikes at 2 dpi with *Fg.* All values are the means ± SE (*n = *3). Asterisks above the bars indicate values that are significantly different from Bw (*P* < 0.05; *t*‐test). (C) Deoxynivalenol (DON) content (µg/g seed) in *Fg‐*inoculated wheat cv. Bw and the *Ubi:WRKY29* transgenic wheat lines. All values are the means ± SE (*n* = 3). Each sample included 3–5 g of seeds collected from two to three spikes derived from separate plants. Asterisks above the bars indicate values that are significantly different from Bw (*P* < 0.05; *t*‐test).

**Figure 6:**
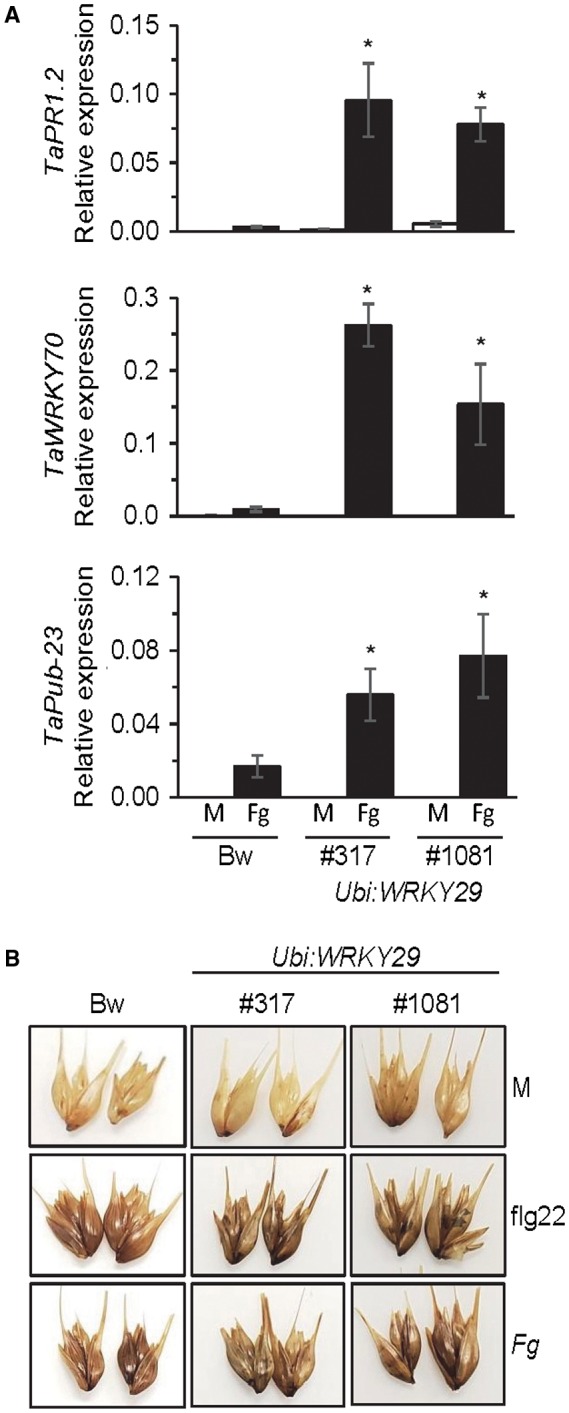
Impact of ectopic *WRKY29* expression on wheat defence responses. (A) Real‐time reverse transcription‐polymerase chain reaction (RT‐PCR) evaluation of *TaPR1.2*, *TaWRKY70 *and *TaPUB23*‐like gene expression in mock (M) and *Fusarium graminearum *(*Fg*)‐inoculated spikes of the wheat cv. Bobwhite (Bw) and *Ubi:WRKY29* transgenic wheat lines #317 and #1081. Gene expression was monitored relative to expression of the control gene *TaTUB2*. All values are the means ± standard error (SE) (*n* = 3). Asterisks above the bars indicate values that are significantly different from Bw (*P* < 0.05; *t*‐test). (B) 3,3′‐Diaminobenzidine (DAB) staining for H_2_O_2_ accumulation in mock (M)‐, flg22‐ and *Fg*‐treated spikelets of the wheat cv. Bw and two independent *Ubi:WRKY29* transgenic lines #317 and #1081. In (A) and (B), spikelets for RNA extraction and DAB staining were harvested at 48 h post‐inoculation (hpi).

To study the feasibility of targeting *WRKY29* expression for the engineering of FHB resistance in wheat, we developed transgenic wheat plants containing a chimeric *Ubi:WRKY29* construct, which constitutively expresses the Arabidopsis *WRKY29* coding sequence (CDS) from the maize *Ubiquitin *promoter. Three independently derived *Ubi:WRKY29* transgenic lines that stably express *WRKY29* were identified (Fig. 5A). All three lines showed significantly higher level of resistance to FHB compared with the control cv. Bobwhite. Disease spread and fungal growth were restricted in the *Ubi:WRKY29* plants compared with the non‐transgenic Bobwhite (Fig. 5A,B). In addition, the accumulation of the mycotoxin DON was also significantly lower in the transgenic *Ubi:WRKY29* lines than in the control cv. Bobwhite (Fig. 5C). Basal expression of the SA‐responsive *TaPR1.2*, as well as the PTI marker genes *TaWRKY70 *and *TaPUB‐23*‐like (Kage *et al*., 2017; Schoonbeek *et al*., 2015), was very low and not altered in the *Ubi:WRKY29* plants compared with the non‐transgenic Bobwhite (Fig. 6A), thus suggesting that, as in Arabidopsis, constitutive expression of *WRKY29* is not sufficient to constitutively activate PTI. However, in response to *Fg* infection, *WRKY29* expression conferred strong expression of *TaPR1.2*, *TaWRKY70* and *TaPUB‐23*‐like in the *Ubi:WRKY29* relative to non‐transgenic Bobwhite plants (Fig. 6A). In contrast, as in Arabidopsis, constitutive expression of *WRKY29* in wheat did not promote stronger H_2_O_2_ accumulation in response to fungal infection (Figs 6B and S2B).

**Figure 7:**
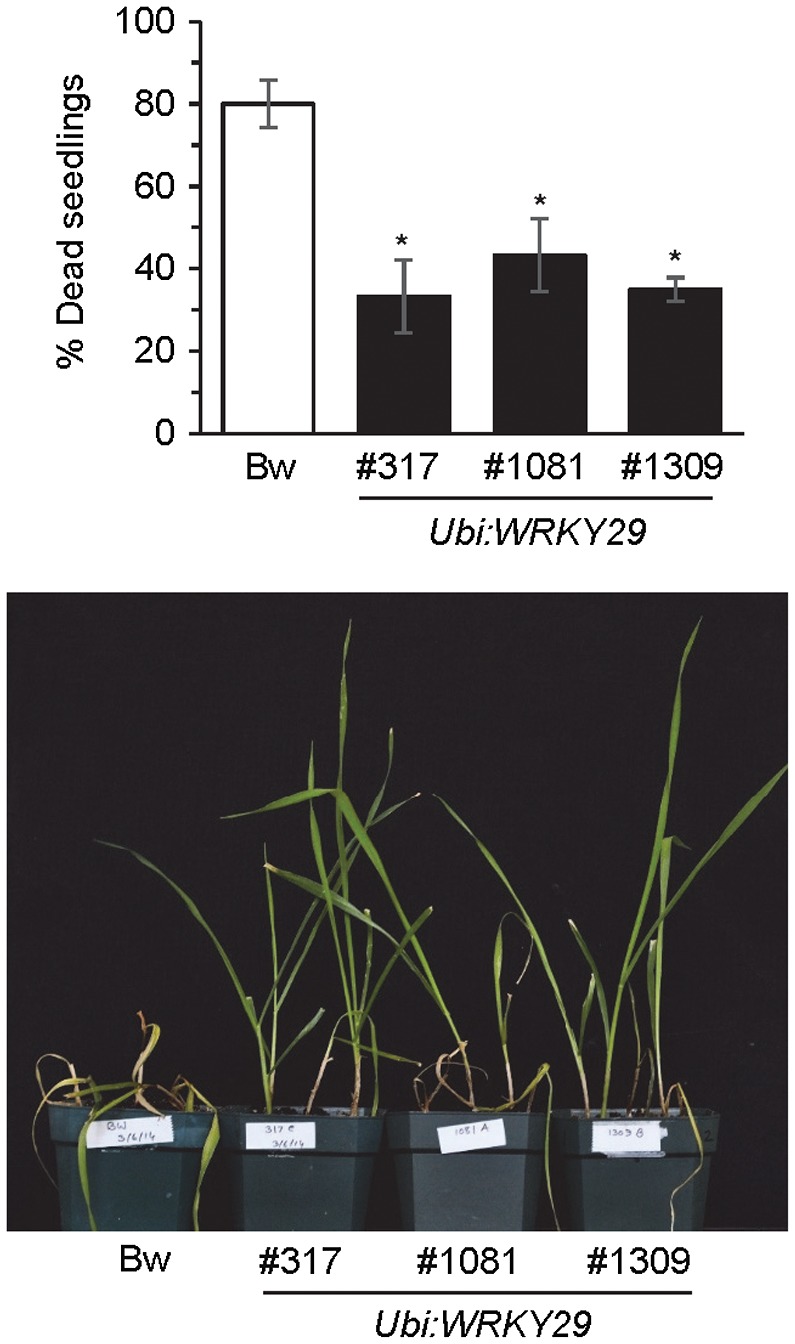
*WRKY29 *expression in wheat promotes resistance to Fusarium seedling blight. Top: percentage of dead seedlings at 2 weeks post‐inoculation of seedlings of wheat cv. Bobwhite (Bw) and three *Ubi:WRKY29* transgenic lines with *Fusarium graminearum. *All values are the means ± standard error (SE) (*n* = 20). Asterisks above the bars indicate values that are significantly different from Bw (*P* < 0.05; *t*‐test). Bottom: photograph showing phenotype in representative seedlings of *F. graminearum‐*inoculated wheat cv. Bw and the *Ubi:WRKY29* transgenic wheat lines. The photograph was taken at 10 days post‐inoculation (dpi).

The *Ubi:WRKY29* plants also demonstrated elevated resistance to Fusarium seedling blight disease (Fig. 7). Taken together, the above results validate our suggestion that the PTI mechanism provides an excellent target for enhancing plant resistance against *Fg*.

### Impact of constitutive *WRKY29* expression on agronomic and growth parameters of wheat

**Figure 8:**
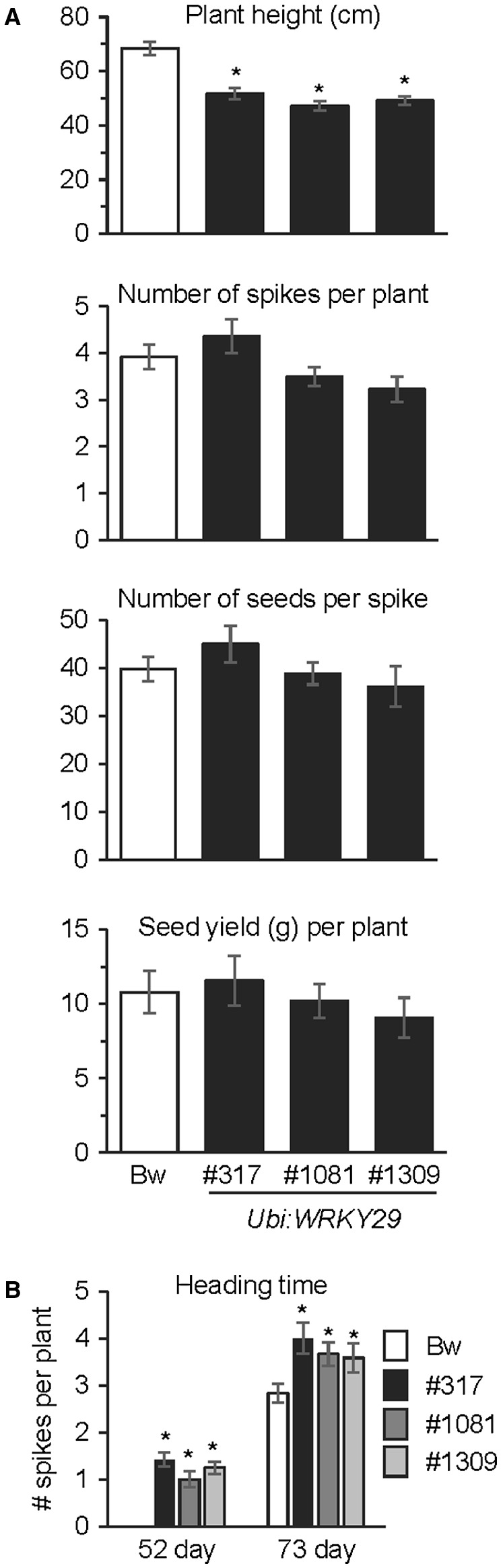
Agronomic and growth parameters in wheat plants expressing *WRKY29*. (A) Comparison of the average plant height (cm), number of spikes per plant, number of seeds per spike and seed yield (g) per plant for the wheat cv. Bobwhite (Bw) and three independent *Ubi:WRKY29* transgenic lines. A minimum of 10 plants per line were analysed to obtain data on plant height, spike numbers and seed yield. The number of seeds per spike was calculated for 10 spikes from each line. (B) Heading time differences between *Ubi:WRKY29* transgenic wheat and the non‐transgenic cv. Bw. The number of spikes in each plant was determined at 52 and 73 days after sowing of the seeds. In (A) and (B), all values are the means ± standard error (SE). Asterisks above the bars indicate values that are significantly different from Bw (*P* < 0.05; *t*‐test).

As shown in Fig. 8A, the *Ubi:WRKY29* wheat plants were 25% shorter than the non‐transgenic Bobwhite plants. This was paralleled by an earlier heading time in the *Ubi:WRKY29* plants compared with Bobwhite (Fig. 8B). No significant impact on other agronomic parameters, such as the number of spikes produced per plant, the number of seeds produced per spike and seed yield per plant, was observed in *Ubi:WRKY29* wheat compared with the control Bobwhite plants (Fig. 8A).

## Discussion

In the absence of monogenic gene‐for‐gene resistance, current control measures for FHB in wheat and barley involve the use of partially resistant varieties combined with fungicide application and management practices (Bai and Shaner, 2004; Wilson *et al*., 2017). Limited knowledge of plant mechanisms that can be targeted to enhance FHB resistance has constrained progress on the development of FHB‐resistant wheat and barley varieties. Previously, using an approach that utilized the interaction between Arabidopsis and *Fg* to identify genes and mechanisms that contribute to defence against *Fg*, and transgenic validation of the ability of these genes/mechanisms to control *Fg* infection in Arabidopsis and wheat, we showed that the SA signalling pathway provides a target for enhancing FHB resistance (Makandar *et al*., 2006, 2010, 2015, 2012). Recent studies have confirmed that genes associated with SA biosynthesis in barley and alleles at *NPR1*‐like genes in winter wheat are associated with basal resistance to FHB (Diethelm *et al*., 2014; Hao *et al*., 2018). Utilizing a similar approach, here we demonstrate that the PTI pathway provides a target for enhancing resistance against *Fg*. We show that resistance against *Fg* can be enhanced in wheat and Arabidopsis by the bacterial MAMP flg22. In Arabidopsis, the flg22‐conferred resistance to *Fg* required the PTI‐associated PRR FLS2. Resistance against *Fg* was also enhanced in wheat and Arabidopsis by the constitutive expression of *WRKY29, *which encodes a transcription factor that is associated with PTI in Arabidopsis. Expression of the Arabidopsis *WRKY29* CDS also restricted DON accumulation in *Ubi:WRKY29* transgenic wheat. Compared with the non‐transgenic Bobwhite, the *Ubi:WRKY29* wheat plants responded to *Fg *infection with stronger expression of *TaPR1.2 *and the PTI marker genes *TaWRKY70* and *TaPUB‐23*‐like, thus suggesting stronger activation of PTI responses. These results, when considered together with the results of Kage *et al*. (2017), who showed that the *TaWRKY70* gene contributes to basal resistance to FHB, demonstrate that the PTI pathway can be targeted to enhance resistance against *Fg*.

The fact that *Fg* infection stimulates *WRKY29* expression to comparably high levels in the WT and *fls2* mutant suggests that another pathway, presumably involving another PRR and its cognate ligand, stimulates PTI leading to *WRKY29 *expression in *Fg*‐inoculated Arabidopsis. The *Fg* infection‐derived elicitor that induces *WRKY29* could potentially be chitin. Previously, a chitooligosaccharide was shown to induce *WRKY29 *expression in Arabidopsis and to promote resistance against fungal and bacterial pathogens (Wan *et al*., 2008). Chitosan treatment also promoted resistance to seed‐borne *Fg* infection in wheat (Bhaskara Reddy *et al*., 1999). Chitosan promoted the accumulation of lignin precursors and phenolics that have antimicrobial activity and could potentially contribute to resistance (Bhaskara Reddy *et al*., 1999). Chitin also induced the expression of wheat homologues of PTI‐associated genes (Schoonbeek *et al*., 2015). More recently, a metabolo‐transcriptomic approach in barley identified *HvCERK1*, a predicted chitin elicitor receptor kinase encoding gene, to be involved in defence against FHB (Karre *et al*., 2017). In Arabidopsis, *WRKY29* expression is also induced by the *Fusarium* T‐2 toxin and other type A trichothecenes (Nishiuchi *et al*., 2006). These toxins, or derived metabolites, could also potentially function as elicitors of PTI in plants infected with *Fg*.

*WRKY *genes include a large family of plant‐specific DNA‐binding proteins that contain the conserved WRKYGQK sequence together with a zinc‐finger‐like motif (Eulgem *et al*., 2000; Pandey and Somssich, 2009). Several of these WRKY proteins are involved in the stress response, including plant defence against pathogens. Although some are positive regulators, others are negative regulators of the stress response (Pandey and Somssich, 2009). In Arabidopsis, *WRKY29 *is associated with PTI and defence against pathogens (Asai *et al*., 2002; Wan *et al*., 2008). Our results indicate an important role for *WRKY29* in the control of *Fg *infection. Not only was *WRKY29* expression up‐regulated in response to *Fg *infection, but, compared with the WT, *Fg* disease severity was higher in the *wrky29* mutant and lower in plants constitutively expressing *WRKY29*. As Arabidopsis *WRKY29* was capable of similarly enhancing disease resistance in wheat, we propose that wheat contains the downstream machinery that is regulated by WRKY29. *WRKY* homologues are present in wheat and barley, and some of these *WRKY*s have been shown to confer stress tolerance when constitutively expressed in heterologous systems (Liu *et al*., 2014; Niu *et al*., 2012; Wang *et al*., 2017, 2015). Recently, a wheat *WRKY* gene, *TaWRKY70 *(also known as *TaWRKY45*), was shown to be required for basal resistance to FHB (Kage *et al*., 2017). *TaWRKY70* is associated with *QTL‐2DL*, which limits FHB severity by the control of fungal spread from the site of initial infection (Kage *et al*., 2017).* TaWRKY70* expression is up‐regulated in FHB‐resistant near‐isogenic lines (NILs) compared with susceptible NILs. *TaWRKY70* expression is also up‐regulated in response to infections with *Puccinia triticina*, which causes leaf rust, and the powdery mildew fungus *Blumeria graminis* (Bahrini *et al*., 2011b). Wheat plants overexpressing *TaWRKY70* exhibit enhanced resistance to FHB, powdery mildew and leaf rust (Bahrini *et al*., 2011a, b), thus suggesting that *TaWRKY70* is involved in defence against a variety of fungal infections. *TaWRKY70* has been suggested to regulate the expression of genes involved in the synthesis of metabolites that are associated with resistance to fungi (Kage *et al*., 2017). Our results indicate that Arabidopsis *WRKY29*, when expressed in wheat, promotes the stronger activation of the PTI pathway, leading to the expression of *TaWRKY70*.

*FLS2* is not required for the *Fg*‐induced up‐regulation of *WRKY29* or for the *flg22*‐induced resistance to *Fg* in Arabidopsis. Furthermore, the Arabidopsis leaf disease index, which reflects the average of disease severity across the different leaf disease categories, was not significantly different between the *Fg*‐inoculated WT and *fls2* mutant, thus suggesting that *FLS2* does not have a major contribution to basal resistance to *Fg *in Arabidopsis. However, a significant difference (*P* < 0.05; *χ* ^2^ test) was observed between the WT and *fls2 *mutant for the relative distribution of leaves over the four disease categories, suggesting a subtle impact of *FLS2* on basal resistance to *Fg*. Plant‐associated microbes are known to prime plant defences (Conrath *et al*., 2015; Pieterse *et al*., 2014). This priming of *FLS2*‐dependent defences by random plant‐associated microbes may influence basal resistance to* Fg *in Arabidopsis.

The results presented here, taken together with the knowledge that *TaWRKY70* and the LysM domain‐containing *HvCERK1 *are required for basal resistance to FHB in wheat and barley, respectively (Kage *et al*., 2017; Karre *et al*., 2017), lead us to propose that genes associated with the PTI pathway are good candidates for the development of FHB‐resistant wheat and barley. Alternatively, factors that can induce the PTI pathway could also promote FHB resistance. However, the impact of PTI pathway activation needs to be tested on additional biotypes of *Fg* to determine whether it is effective against the different chemotypes of *Fg*, as well as other FHB‐causing *Fusarium* species. *Ubi:WRKY29* wheat also exhibits reduced plant height and faster heading time, without any detrimental effects on yield. Height and heading time are traits that are important to wheat breeding (Hedden, 2003; Wilhelm *et al*., 2013). Thus, the pathway targeted by *WRKY29* has the potential to influence additional beneficial traits for wheat breeding.

## Experimental Procedures

### Cultivation of Arabidopsis and wheat

A peat‐based soil mix (Fafard #2, Sungro, Agawam, MA) was used to cultivate Arabidopsis and wheat. Arabidopsis was cultivated as described previously (Nalam *et al*., 2016) in growth chambers programmed for 22 ºC and a 14‐h light (80–100 µE/m^2^/s) and 10‐h dark regime. The soil was autoclaved for 1 h prior to use. Arabidopsis *npr1‐1*, *wrky29* (CS3024690) and *fls2‐101* mutants, and the *35S:NPR1* transgenic lines and the wheat *Ubi:NPR1* transgenic line in the cultivar Bobwhite, have been described previously (Cao *et al*., 1998; Li *et al*., 2017; Makandar *et al*., 2006; Pfund *et al*., 2004). Generation of the Arabidopsis *35S:WRKY29* and *35S:PR1‐flg22* lines and wheat *Ubi:WRKY29* lines is described below. Wheat was cultivated in a glasshouse in which natural sunlight was supplemented with halogen lamps to provide a minimum of 14 h exposure to light. The glasshouse was programmed for day/night‐time temperatures of 21 ºC and 18 ºC, respectively.

### Pathogen strains, culture conditions and plant infection

Half‐strength potato dextrose medium (Difco Laboratories, Detroit, MI, USA) was used for the growth and maintenance of *Fg *isolate Z‐3639, and carboxymethylcellulose (CMC) medium was used to promote sporulation, as described previously (Nalam *et al*., 2016). The fungus was cultivated at 28 °C. Fungal inoculation of Arabidopsis leaves involved infiltration of a suspension of fungal mycelial fragments through the abaxial surface with a needleless syringe (Nalam *et al*., 2016). Approximately 4‐week‐old Arabidopsis plants were used for leaf assays and 6–7‐week‐old plants for inflorescence assays. Disease severity was scored at 5 dpi, unless stated otherwise. Depending on the extent of chlorosis, leaves were grouped into four categories: Category I, chlorosis covering <25% of leaf area; Category II, chlorosis covering 25%–50% of leaf area; Category III, chlorosis covering 50%–75% of leaf area; Category IV, chlorosis covering 75%–100% of leaf area. A minimum of 50 leaves of each genotype/treatment were analysed for each experiment. The percentage of leaves in each category was used to calculate the leaf disease index, as described previously (Nalam *et al*., 2016). Inoculations of Arabidopsis inflorescences with *Fg* macroconidia and the disease rating [expressed as the Fusarium Arabidopsis Disease (FAD) score] were conducted as described previously (Nalam *et al*., 2016).

Inoculation of wheat spikelets with *Fg* macroconidia and disease evaluation were performed as described previously (Makandar *et al*., 2006, 2012). Briefly, at the anthesis stage, two central spikelets were inoculated with 10 µL of a suspension containing 300 fungal macroconidia. High humidity was maintained for 3 days by covering the inoculated spikes with a moistened zip‐lock bag. Over time, the fungal infection spread out to the other spikelets within each spike. Disease spread was monitored at periodic intervals. The final reading was taken at 21 dpi and the disease severity was calculated as the percentage of diseased spikelets.

To study the effect of wheat genotypes on Fusarium seedling blight, seeds of the indicated lines were soaked with an *Fg* macroconidial suspension for 24 h. In addition, after germination, seedlings were spray inoculated with a macroconidial suspension (100 000 macroconidia/mL) and covered for 3 days. The percentage of surviving seedlings was determined at 14 dpi.

### flg22 peptide treatment

A needleless syringe was used to infiltrate 50 ng of flg22 peptide (QRLSTGSRINSAKDDAAGLQIA; Alpha Diagnostic, International Inc., San Antonio, TX; Cat# FLG22‐p‐1) dissolved in 20 µL of water through the abaxial surface of Arabidopsis leaves. Water‐infiltrated leaves provided the controls. After 24 h, the leaves were harvested for RNA isolation or treated with *Fg* mycelial fragments to monitor the impact of PTI activation on fungal disease. For experiments with wheat, flg22 peptide (50, 100 and 200 ng), dissolved in 10 µL of water, and water as control, were applied with a Hamilton syringe to the central spikelet of each spike.

### Mycotoxin analysis

DON content in wheat grains was determined as described previously (Fuentes *et al*., 2005; Mirocha *et al*., 1998).

### Arabidopsis and wheat transgenics

The *35S:NPR1* Arabidopsis and *Ubi:NPR1* wheat plants used in this study have been described previously (Cao *et al*., 1998; Makandar *et al*., 2006). Three PCR steps were used to generate the *PR1‐flg22* chimera, such that the flg22 peptide fused to the apoplast‐localized PR1 protein could be delivered to the extracellular space. In the first PCR, cDNA prepared from Arabidopsis leaves was used to amplify the *PR1* CDS (At2g14610) with the primers PR1‐CDS‐F(*Bam*H1) and flg22(21)‐AtPR1(12)‐R to give a 513‐bp amplicon containing the PR1 CDS, without the stop codon, followed by the coding information for the first seven amino acids of flg22. In a second PCR, the flg22‐F and flg22‐R 66‐mer oligos, which contain the coding information for the flg22 peptide (QRLSTGSRINSAKDDAAGLQIA), were mixed with the PR1(21)‐flg22(12)‐F and flg22‐R(*Cla*I) primers (Table S1, see Supporting Information) which, on PCR, yielded a 99‐bp product which, at one end, contained the information for the last seven amino acids of PR1 fused to flg22 with a stop codon included at the end of the flg22 CDS. The products of the previous two PCRs were mixed in equal proportions and used in PCR with the primers PR1‐CDS‐F(*Bam*H1) and flg22‐R(*Cla*I) (Table S1) to yield a 570‐bp product that includes the PR1 CDS fused at its C‐terminal end to the flg22 CDS with a stop codon included. This final product was cloned into the pCR8/GW/TOPO entry vector (Life Technologies, Carlsbad, CA; www.lifetechnologies.com) from which the insert was mobilized into the destination binary vector pMDC32 with the Gateway LR clonase system (Life Technologies; www.lifetechnologies.com). The resultant pMDC32:*PR1‐flg22* plasmid contains the *PR1‐flg22* chimera between the *Cauliflower mosaic virus 35S* promoter and *Agrobacterium tumefaciens nos* (*nopaline* *synthase*) gene terminator. This construct was transformed into Arabidopsis accession Columbia and the *fls2* mutant by the floral dip method (Zhang *et al*., 2006). Hygromycin‐resistant transformants containing the *35S:PR1‐flg22* chimera were selected in the presence of hygromycin (25 mg/L). The presence of the insert was confirmed by PCR and expression was tested by RT‐PCR.

The *WRKY29* (At4g23550) CDS was amplified from cDNA prepared from flg22‐inoculated leaves with the primers WRKY29‐CDS‐F(*Bam*HI) and WRKY29‐CDS‐R(*Cla*I) (Table S1). The resultant amplicon was cloned into the pMDC32 vector via the pCR8/GW/TOPO intermediate, as described above for *35S:PR1‐flg22*. The resultant plasmid pMDC32‐*WRKY29* contains the *WRKY29* CDS flanked on its 5′ end by the *35S* promoter and at the 3′ end by the *nos* terminator. This construct was transformed into Arabidopsis accession Columbia by the floral dip method (Zhang *et al*., 2006). Hygromycin‐resistant transformants were selected as described above for *35S:PR1‐flg22*. The presence of the *35S:WRKY29* transgene in Arabidopsis was monitored by PCR conducted with 35S‐F and WRKY29‐R(*Cla*I) primers (Table S1).

The *Ubi*:*WRKY29* chimeric gene was generated by cloning the *WRKY29* CDS amplicon, generated as described above, in the pJS406 backbone (Makandar *et al*., 2006). The resultant pSS:*Ubi*:*WRKY29* plasmid contains the *WRKY29* CDS flanked on the 5′ end by the maize *Ubi* gene promoter plus intron (Christensen and Quail, 1996) and on the 3′ end by the *nos* terminator. To generate transgenic wheat, the pSS:*Ubi:WRKY29 *plasmid and the *bar* selectable marker containing plasmid pAHC20 (Christensen and Quail, 1996) were co‐bombarded into embryos from the spring wheat cv. Bobwhite, and wheat plants were regenerated as described previously (Anand *et al*., 2003). Glufosinate (Liberty; Bayer Crop Sciences, Research Triangle, NC, USA) resistance conferred by the *bar* gene was used as the selectable marker for transgenic wheat. The presence of the *Ubi:WRKY29* transgene was monitored by PCR using the WRKY29‐F and WRKY29‐R primers (Table S1). PCR conditions included a 5‐min denaturation at 95 ºC, followed by 50 cycles of 95 ºC for 45 s, 55 ºC for 45 s and 72 ºC for 60 s, with a final extension of 72 ºC for 7 min.

### DNA and RNA isolation

DNA for PCR and genotyping was extracted from leaf tissue as described previously (Nalam *et al*., 2016). RNA was extracted from frozen tissues using an acidic guanidinium thiocyanate–phenol–chloroform mix (Chomczynski and Sacchi, 1987).

### RT‐PCR and real‐time PCR

After removal of DNA with RQ1 RNase‐free DNase (Promega, Madison, WI, USA), the purified RNA was used for cDNA synthesis with oligo‐dT 18‐mer primer (New England Biolabs, Ipswich, MA, USA) and GoScript™ reverse transcriptase (Promega). The cDNA was subsequently utilized for RT‐PCR and quantitative real‐time RT‐PCR.

The primer pairs WRKY29‐qRT‐F plus WRKY29‐qRT‐R, and EF‐qRT‐F plus EF‐qRT‐R (Table S1), were used for RT‐PCR to monitor the expression of Arabidopsis *WRKY29* and At1g07940, respectively. At1g07940, which encodes an elongation factor related to EF‐1α, was previously identified as a gene that is very suitable for the normalization of gene expression (Czechowski *et al*., 2005). The PCR conditions for *WRKY29* included a 3‐min denaturation at 95 ºC, followed by 25 cycles of 95 ºC for 30 s, 58 ºC for 30 s and 72 ºC for 30 s, with a final extension of 72 ºC for 5 min. The PCR conditions for At1g07940 included a 3‐min denaturation at 95 ºC, followed by 25 cycles of 95 ºC for 30 s, 55 ºC for 30 s and 72 ºC for 30 s, with a final extension of 72 ºC for 5 min. RT‐PCR was used to monitor the expression of *PR1‐flg22* from the *35S:PR1‐flg22* construct in Arabidopsis. The primer pairs PR1‐CDS‐F(*Bam*HI) and flg22‐R(*Cla*I) (Table S1) were used in the PCR. Expression of the Arabidopsis *ACT8* gene was monitored as control with the primer pair ACT8‐RT‐F plus ACT8‐RT‐R (Table S1). The PCR conditions included a 3‐min denaturation at 95 ºC, followed by 30 cycles of 95 ºC for 30 s, 55 ºC for 30 s and 72 ºC for 45 s, with a final extension of 72 ºC for 7 min. To monitor *WRKY29* expression from the *Ubi:WRKY29* construct in wheat, the primer pairs WRKY29‐F plus WRKY29‐R (Table S1) were used for amplification in the RT‐PCRs. Expression of the wheat *TaTUBB2 *(*Tubulin beta‐2*) gene was used as control for RT‐PCR. The primer pair TaTUBB2‐F and TaTUBB2‐R (Table S1) was used in the PCR. The PCR conditions for *WRKY29* expression derived from the *Ubi:WRKY29* chimera in wheat included a 3‐min denaturation at 95 ºC, followed by 40 cycles of 95 ºC for 30 s, 60 ºC for 30 s and 72 ºC for 40 s, with a final extension of 72 ºC for 5 min. The PCR conditions for *TUBB2 *included a 3‐min denaturation at 95 ºC, followed by 40 cycles of 95 ºC for 30 s, 58 ºC for 30 s and 72 ºC for 30 s, with a final extension of 72 ºC for 5 min.

Quantitative real‐time RT‐PCR was performed with Sybr Green PCR Master Mix (Bio‐Rad Laboratories, Hercules, CA, USA) on an Eco qPCR system (Illumina, San Diego, CA) using the following amplification conditions: 10‐min polymerase activation and denaturation at 95 °C, 40 cycles of 95 °C for 10 s, 58 °C for 30 s and 72 °C for 30 s. This was followed by a product melt to confirm a single PCR product. The level of individual gene expression was normalized to that of At1g07940 by subtracting the cycle threshold value for At1g07940 from the cycle threshold value of the test genes. Fold induction, when calculated, was relative to expression in the mock‐treated plants. The primer pairs WRKY29‐qRT‐F plus WRKY29‐qRT‐R, and EF‐qRT‐F plus EF‐qRT‐R (Table S1), were used for real‐time RT‐PCR to monitor the expression of Arabidopsis *WRKY29* and At1g07940, respectively. For qRT‐PCR analysis of wheat *TaPR1.2*, *TaWRKY70* and *TaPUB‐23*‐like, the primer pairs TaPR1.2‐F plus TaPR1.2‐R, TaWRKY70‐F plus TaWRKY70‐R, and TaPUB‐23‐F plus TaPUB‐23‐R, respectively, were used (Table S1). The expression of these genes was normalized to that of *TaTUBB2*.

### Histological examination for H_2_O_2_ accumulation

*In situ* accumulation of H_2_O_2_ was monitored by staining leaves with 3,3′‐diaminobenzidine (DAB; Sigma‐Aldrich, St Louis, MO, USA) using a protocol developed by Thordal‐Christensen *et al*. (1997) as modified by Gao X *et al*. (2013). Briefly, Arabidopsis leaves were infiltrated with *Fg*, whereas wheat leaves were first pierced with a needle and *Fg* spores were placed on the pierced site. Leaves infiltrated with water and untreated leaves provided the controls. The treated plants were left in the growth room until ready for harvest. On harvest, leaves were immersed in DAB solution (1 mg/mL; pH 3.8). Gentle vacuum was applied for 20 min to infiltrate the DAB solution into the leaves. Leaves immersed in DAB solution were covered in foil and incubated at room temperature. After 8 h, the DAB solution was discarded. For destaining, Arabidopsis leaves were boiled in 95% ethanol for 20 min, whereas wheat leaves were boiled for 30 min. Destained leaves were stored in 70% ethanol. A similar process was used for staining wheat spikes, except that the destaining utilized 70% ethanol and was conducted overnight in a shaker at 70 °C. The destained leaves were observed under a light microscope.

### Statistical analysis

Two‐tailed Student’s *t*‐test was used to determine the significance of variance (*P* < 0.05) when comparing two treatments or genotypes. The *χ* ^2^ test was used to determine whether the differences between disease categories on Arabidopsis leaves were significantly different (*P* < 0.05) between two genotypes and treatments. Analysis of variance (ANOVA) following the General Linear Model, followed by Tukey’s multiple comparison test, was used to determine the significance of variance (*P* < 0.05; Minitab v15; www.minitab.com) when comparing multiple genotypes and/or treatments with each other.

### Accession numbers

At4g23550 (Arabidopsis *WRKY29*), At5g46330 (Arabidopsis *FLS2*), At1g07940 (Arabidopsis GTP‐binding Elongation factor Tu family), At2g14610 (Arabidopsis *PR1*), At1g49240 (Arabidopsis *ACT8*), U76745 (wheat *TUBB2*), AJ007349 (wheat *PR1.2*), BQ743320 (wheat *PUB‐23*‐like), AB603890 (wheat *WRKY70*), FGSG_0811 (*Fg* *NahG*).

## Conflicts of Interest

The authors declare that there are no conflicts of interest.

## Supporting information


**Fig. S1  **Expression of the *PR1‐flg22* chimera in transgenic Arabidopsis. Top: reverse transcription‐polymerase chain reaction (RT‐PCR) analysis of the *PR1‐flg22* chimeric transcript and, as control, the Arabidopsis *ACT8 *gene in the wild‐type (WT) accession Columbia and two independent *PR1‐flg22* lines #2 and #5 in the *FLS2* background. Bottom: *PR1‐flg22* and *ACT8* expression in the *fls2* mutant and two independent *PR1‐flg22* lines #2 and #5 that are in the *fls2* mutant background.

**Fig. S2  **3,3′‐Diaminobenzidine (DAB) staining for H_2_O_2_ accumulation in *Fusarium graminearum‐*inoculated Arabidopsis and wheat leaves. (A) H_2_O_2_ accumulation in *F. graminearum‐*inoculated leaves of wild‐type (WT) accession Columbia and three independent *35S:WRKY29* transgenic Arabidopsis lines. (B) H_2_O_2_ accumulation in *F. graminearum‐*inoculated leaves of wheat cv. Bobwhite (Bw) and two independent *Ubi:WRKY29* transgenic lines #317 and #1081. In (A) and (B), leaves were harvested for DAB staining at 2 and 6 h post‐inoculation.

**Table S1  **Primers used in this study.
