# Supplementary figures and images for: Targeting the pattern‐triggered immunity pathway to enhance resistance to Fusarium graminearum
Source: Mol Plant Pathol. 2019 Feb 6;20(5):626–40. doi: 10.1111/mpp.12781 (PMC6637896; doi:10.1111/mpp.12781)

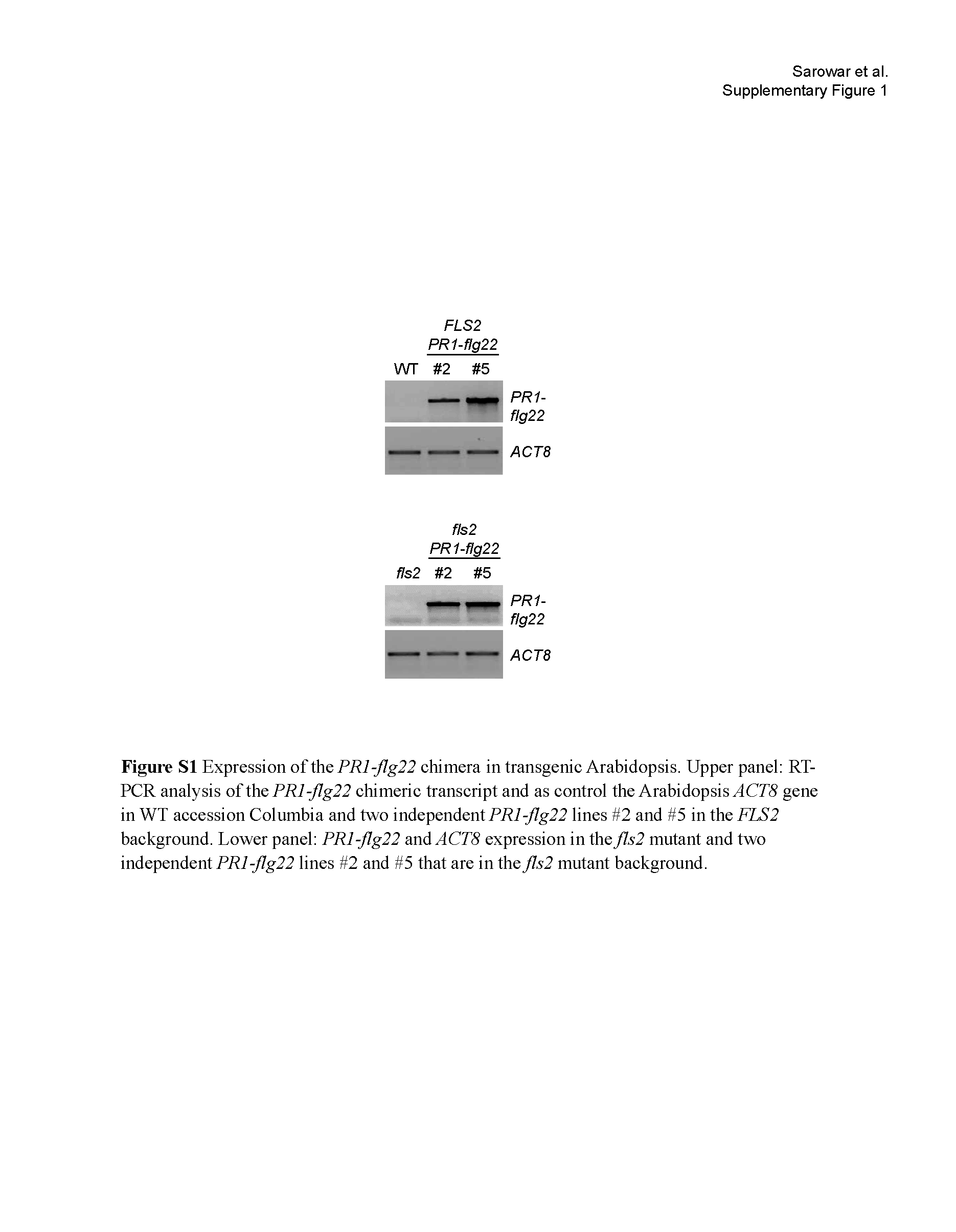

Supplement: Supplementary file 1 — Fig. S1 Expression of the PR1‐flg22 chimera in transgenic Arabidopsis. Top: reverse transcription‐polymerase chain reaction (RT‐PCR) analysis of the PR1‐flg22 chimeric transcript and, as control, the Arabidopsis ACT8 gene in the wild‐type (WT) accession Columbia and two independent PR1‐flg22 lines #2 and #5 in the FLS2 background. Bottom: PR1‐flg22 and ACT8 expression in the fls2 mutant and two independent PR1‐flg22 lines #2 and #5 that are in the fls2 mutant background. [file MPP-20-626-s001.tiff]

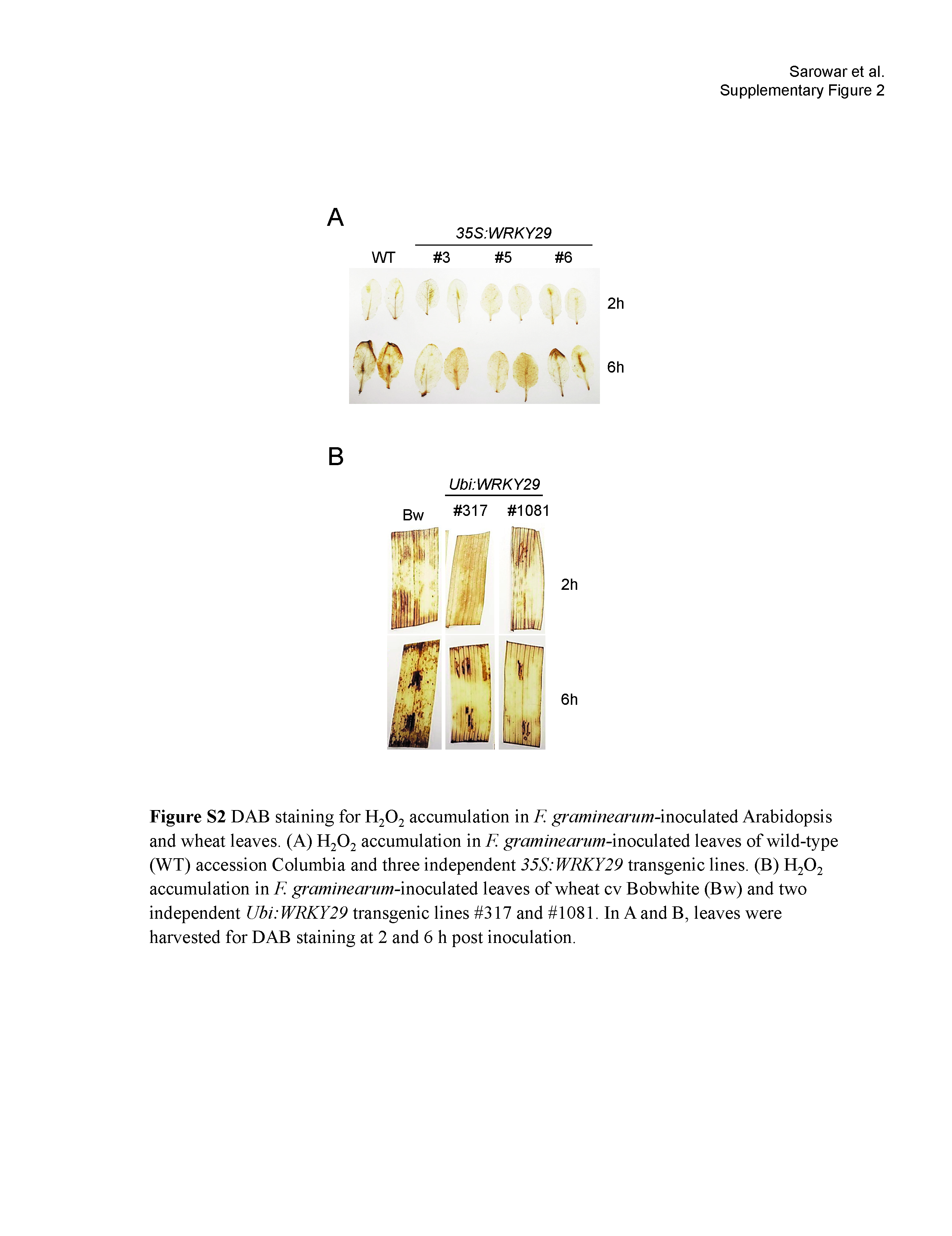

Supplement: Supplementary file 2 — Fig. S2 3,3′‐Diaminobenzidine (DAB) staining for H2O2 accumulation in Fusarium graminearum‐inoculated Arabidopsis and wheat leaves. (A) H2O2 accumulation in F. graminearum‐inoculated leaves of wild‐type (WT) accession Columbia and three independent 35S:WRKY29 transgenic Arabidopsis lines. (B) H2O2 accumulation in F. graminearum‐inoculated leaves of wheat cv. Bobwhite (Bw) and two independent Ubi:WRKY29 transgenic lines #317 and #1081. In (A) and (B), leaves were harvested for DAB staining at 2 and 6 h post‐inoculation. [file MPP-20-626-s002.tiff]
