# Supplementary material for: Targeting the pattern‐triggered immunity pathway to enhance resistance to Fusarium graminearum
Source: Mol Plant Pathol. 2019 Feb 6;20(5):626–40. doi: 10.1111/mpp.12781 (PMC6637896; doi:10.1111/mpp.12781)
Supplement: Supplementary file 3 — Table S1 Primers used in this study. [file MPP-20-626-s003.docx]

**Table S1** Primers used in this study.

| Primer Name | Organism | Gene ID | Purpose | Primer Sequence (5’🡪3’) |
| --- | --- | --- | --- | --- |
| ACT8-RT-F | *A. thaliana* | At1g49240 | RT-PCR | AGCCAGATCTTCATCGTCGT |
| ACT8-RT-R | *A. thaliana* | At1g49240 | RT- PCR | CAACAACACTGGGGAAAACC |
| PR1-CDS-F(*Bam*HI) | *A. thaliana* | At2g14610 | Cloning | CGCGGATCCATGAATTTTACTGGC |
| Flg22-F | *P. aeruginosa* |  | Cloning | CAGCGCCTGAGCACCGGCAGCCGCATTAACAGCGCGAAAGATGATGCGGCGGGCCTGCAGATTGCG |
| Flg22-R | *P. aeruginosa* |  | Cloning | CGCAATCTGCAGGCCCGCCGCATCATCTTTCGCGCTGTTAATGCGGCTGCCGGTGCTCAGGCGCTG |
| PR1(21)-flg22(12)-F |  |  | Cloning | TATGTGAACGAGAAGCCATACCAGCGCCTGAGC |
| flg22(21)-PR1(12)-R |  |  | Cloning | GCTGCCGGTGCTCAGGCGCTGGTATGGCTTCTC |
| flg22-R(*Cla*I) |  |  | Cloning | CCCATCGATTTACGCAATCTGCAG |
| PR1-qRT-F | *A. thaliana* | At2g14610 | qPCR | CGGAGCTACGCAGAACAACT |
| PR1-qRT-R | *A. thaliana* | At2g14610 | qPCR | CTCGCTAACCCACATGTTCA |
| WRKY29-F(*Bam*HI) | *A. thaliana* | At4g23550 | Cloning | GTGGGATCCATGGACGAAGGAGAC |
| WRKY29-R(*Cla*I) | *A. thaliana* | At4g23550 | Cloning | CCCATCGATCTAGTAATTCCA |
| WRKY29-F | *A. thaliana* | At4g23550 | RT-PCR | ACCCTTTCTCCACACAAACG |
| WRKY29-R | *A. thaliana* | At4g23550 | RT-PCR and PCR | CCGCAACGCTCATTTCTT |
| WRKY29-qRT-F | *A. thaliana* | At4g23550 | qPCR | ATCCAACGGATCAAGAGCTG |
| WRKY29-qRT-R | *A. thaliana* | At4g23550 | qPCR | GATGGGTTTCTGCCCGTATT |
| WRKY70-qRT-F | *A. thaliana* | At3g56400 | qPCR | CCCAAGAAGTTACTTTAGATGCAC |
| WRKY70-qRT-R | *A. thaliana* | At3g56400 | qPCR | TTGCTCTTGGGAGTTTCTGC |
| EF-qRT-F | *A. thaliana* | At1G07940 | RT-PCR | ACCAAGATTGACAGGCGTTC |
| EF-qRT-R | *A. thaliana* | At1G07940 | RT-PCR | TGCAACAGTCTGCCTCATGT |
| TaPR1.2-F | *T. aestivum* | AJ007349 | qPCR | GACTTCGTCAACCTGCACAA |
| TaPR1.2-R | *T. aestivum* | AJ007349 | qPCR | GCCGGAATGTGTGCTTATTT |
| TaPUB23-like-F | *T. aestivum* | BQ743320 | qPCR | CGTTCATCAGAATGCTCAGCTG |
| TaPUB23-like-R | *T. aestivum* | BQ743320 | qPCR | TTCTCTTTTGTAGGCACGAACCA |
| TaTUBB2-F | *T. aestivum* | U76745 | RT-PCR | GAATGCTGATGAGTGCATGG |
| TaTUBB2-R | *T. aestivum* | U76745 | RT-PCR | CGAGGGAATGGGATAAGGTT |
| TaWRKY23-like-F | *T. aestivum* | Traes_1DL_46428511F.1 | qPCR | GAGCGTAGACGTCAGCACCA |
| TaWRKY23-like-R | *T. aestivum* | Traes_1DL_46428511F.1 | qPCR | CACGGATGCTAATGGCCA |
| TaWRKY70-F | *T. aestivum* | AB603890 | qPCR | TCGACGACGCTGCTGCGACG |
| TaWRKY70-R | *T. aestivum* | AB603890 | qPCR | GCAGAGCTCCCTGGCGTGC |
| FgNahG-F | *F. graminearum* | FGSG_08116 | qPCR | GGTGCTGATGGTATTCACTCT |
| FgNahG-R | *F. graminearum* | FGSG_08116 | qPCR | GTCGTGCATGAAGGTAGC |
| 35S-F | CaMV |  | PCR | GGAGAGGACCTCGACTCTAGA |
